# Sliding mode dynamics of a non-smooth Filippov predator-prey system for integrated pest management

**DOI:** 10.1371/journal.pone.0334425

**Published:** 2025-10-23

**Authors:** Juan Liu, Jie Hu, Haiyan Song

**Affiliations:** 1 College of Agricultural Engineering, Shanxi Agricultural University, Taigu, People’s Republic of China; 2 Department of Basic Sciences, Shanxi Agricultural University, Taigu, People’s Republic of China; 3 School of Software, Shanxi Agricultural University, Taigu, People’s Republic of China; National Museums of Kenya Ornithology Section, KENYA

## Abstract

In recent years, the problem of pests seriously affects the yield and quality of crop, posing a major challenge to the safe production of crop, which have seriously hindered the development of China’s agriculture. How to quickly and accurately monitor pests, timely grasp the occurrence dynamics of pests, and prevent and control pests is of great significance for reducing crop yield losses. Considering the discontinuity of spraying pesticides and releasing natural enemies in the process of pest control, and the Filippov system’s ability to accurately depict switching states and human intervention measures, a non-smooth Filippov predator-prey system with threshold strategies is investigated incorporating several different functional responses, such as Holling II functional response and ratio functional response etc, which should be selectively applied dependent on the population of the prey. The aim of this study is to investigate the complex dynamics including bistabilities of the ecosystem when the relative populations of the prey and predator is substantially different, by modelling the non-smooth Filippov system with multiple switchable functional responses for the very first time, which is believed to be more realistic for modeling the dynamics of real ecosystem, thus the solution of the present work may be more suitable for real world applications such as for the integrated pest management. The validity of the proposed system is assessed by simulation, and bifurcation set of equilibria and the global stability of equilibria has been numerically obtained through an arbitrary set of parameters. Moreover, the dynamic behaviors of proposed system, such as the existence of various equilibria and their global stabilities; the existence of various domains such as the sliding domain, escaping domain and crossing domain, have been analyzed in great details in the present work. It is shown that the sliding region and escaping region cannot coexist when the density of the prey and predators is substantially different. It is further demonstrated that the real equilibrium and pseudo-equilibrium points can coexist when the population of the prey is less than that of the predator; and only the virtual equilibrium and pseudo-equilibrium can coexist in the case of when the population of the prey is more than that of the predator.In particular, it is noted that all trajectories of the prey and predators population are eventually converging into certain equilibrium points as it is demonstrated in the numerical simulation. This implies that there exists global asymptotic stability of equilibrium points under the proposed system, in which the population of preys eventually reaches a steady state of density at the real equilibrium and pseudo-equilibrium points. This work also highlights the significant role of the threshold in the process of pest controls: it is seen from this work that different types of equilibrium points can occur dependent on the choice of the economic threshold (ET). The conclusions obtained will be applied to Unmanned Aerial Vehicle (UAV) to spray pesticides and release natural enemies in a timely and quantitative manner, thereby achieving efficient and rapid monitoring and control of large-scale crop. This can more effectively ensure stable and high crop yields, provide theoretical guidance for scientific prevention and control, and is of great significance for reducing the burden on farmers, promoting agricultural development, and realizing agricultural modernization.

## 1 Introduction

In recent years, the outbreak of crop diseases and pests has become more and more frequent, resulting in serious crop losses and even production failures, which have seriously hindered the development of China’s agriculture. A pest is a species that damages other valuable populations or interferes with human activities, so it is necessary to take measures to reduce pest damage to crops [1–5]. In the pest-natural enemy ecosystem, pests and natural enemies are interdependent and mutually restricted, and an appropriate amount of pests can maintain the ecological balance. If the pests are completely killed, the ecological imbalance will be caused, and the natural enemy population will be extinct due to lack of food. As a result, Integrated pest Management (IPM), a threshold control strategy that combines chemistry, economics, and biology, was developed. Integrated pest management (IPM) is implemented to keep pest populations below the economic harm level (EIL) rather than eliminate them completely, which benefits individual cropping systems and local ecosystems [3–5].

Discontinuity in the dynamics of animals’ population in the natural environment seems to be an universal property of the ecosystem, as the animal’s survivals and growth rate are subject to the impact of food resources, climatic conditions, seasonal change and human factors. To understand and to predict when these discontinuities may occur with high degree of accuracy, the systems are needed to be modelled by using non-smooth functional responses. Systems which exhibit non-smooth behavior can be broadly divided into three different types dependent on the degree of smoothness : i) non-smooth continuous system, ii) impulsive system and iii) Filippov system. Tang Sanyi et al. and other experts [1–5] studied the non-smooth continuous pest control under the integrated pest management system, by using the Impulsive Differential Equation to model the spraying of pesticide at fixed time intervals, and to release natural enemies intermittently dependent on the environmental conditions. Similar studies but using a general functional response and impulsive control, had been reported by the authors [3] in 2020 for the study of the extinction and permanence of the predator-prey system. Recent work that employed generalized functional response for modelling the population dynamics in the ecosystem has been further extended by the same authors for three species impulsive system [4], as well as a m-prey and n-predator impulsive system [5] under seasonal disturbance factors, had also been reported. One of the drawbacks for all the previous work has been the modelling of the ecosystem when the control strategy is instantaneously applied, and at the same time to deduce the effects of the control immediately after the control strategy is implemented. This methodology has modelled the reduction of pest population over a short period of time after the control strategy is applied, which may over estimate the number of pest deaths than it would actually happen in practice. To model closer in line with the real environment, it is necessary to introduce a continuity of control strategy, like the Filippov system [6–20], which allows the monitoring of the increase or the reduction of pest population before and after the control measures have been applied. In recent years, there are many encouraging reports that use Filippov system for pest control such as the work by Tang Sanyi et al. In 2019 Qin et al. [10] investigated the threshold control strategy for a non-smooth Filippov ecosystem which featured a group defense from the pest. In 2021 Arafa et al. [14] studied the effectiveness of population dynamics by using Filippov pest control model which incorporates with a time delay. The global dynamics of the Filippov predator-prey model which featured two independent thresholds for the integrated pest management (IPM) was discussed by Li et al. [8] in 2022. At the same time, Jiao et al. [8] probed the dynamics and bifurcations of the predator-prey system using Filippov Leslie-Gower response function to model the group defense of the pest with time delays.

As far as the authors aware, most if not all of the existing literatures including those mentioned above in [6–21], have assumed a single rate of feeding by the prey, i.e. the predation process is described by a single functional response for the entire period within the predator-prey system. This paper attempts to fill the gap by modelling a non-smooth Filippov predator-prey system with threshold strategies is investigated incorporating several different functional responses for the first time. Different from previous research, the present work develops the modelling for an integrated pest management (IPM) especially with several different functional responses such as Holling II functional response and ratio functional response etc, which should be selectively applied dependent on the population of the prey. In practice, the mutual competition amongst the predator is dependent on its population for a given number of prey in the environment. For example, in the predator-prey system, the thresholds about the preys are related to the population of predators. When the number of preys *x*(*t*) are in abundance, namely *x*(*t*) > *ET* > 0, where ET is the economic threshold, then the predator which has population of *y*(*t*) should not mutually compete for food as the result of sufficient of food for the predators. Thus the interactions of the prey-dependent can be described by functional responses such as Holling-I functional response $\left\{ \begin{array}{ll} \frac{b}{a}x(t) & 0<x(t)<a\\ b & x(t)>a \end{array}\right.$, Holling-II functional response $\frac{rx(t)}{a+bx(t)}$, Holling-III functional response $\frac{rx{\left(t\right)}^{2}}{a+bx{\left(t\right)}^{2}}$ and Ivlev functional response $r\left(1-e^{-\alpha x(t)}\right)$ and so on. As an example we can adopt the Holling-II functional response to obtain the subsystem model of the predator-prey system

$$\left\{ \begin{array}{c} \frac{dx\left(t_{1}\right)}{dt_{1}}=rx(t_{1})(1-\frac{x(t_{1})}{K})-\frac{ax(t_{1})y(t_{1})}{1+bx(t_{1})}\\ \frac{dy\left(t_{1}\right)}{dt_{1}}=-Dy(t_{1})+\frac{ex(t_{1})y(t_{1})}{1+bx(t_{1})} \end{array}\right.$$ (1)

where $x\left(t_{1}\right),y\left(t_{1}\right)$ denote the densities of the prey (pest) and predator (natural enemy) at time *t*_1_, respectively. *r* > 0 is the intrinsic growth rate of the prey, *K* > 0 is the carrying capacity of the prey, *D* > 0 is the death rate of the predator. The *e* > 0 denotes the rate of converting the consumed preys into the growth of predators, and the $\frac{ax(t_{1})y(t_{1})}{1+bx(t_{1})}$ is the Holling-II functional response which represents the rate of predation by the predator per-capita.

While the number of preys is declining to less than a certain multiple of the number of predators *x*(*t*) < *ET*, the mutual interference between predators will be triggered to take effect and the predator-dependent type will dominate the interactions between the predator and prey. In this case functional responses such as ratio-dependent $\frac{ax(t)y(t)}{bx(t)+cy(t)}$, and others like the Beddington-DeAngelis functional response$\frac{mx(t)}{a+bx(t)+cy(t)}$, Watt-type functional response $exp\frac{-cx(t)}{y{\left(t\right)}^{m}}$ and so on are more suitable to model the predator-prey system. The ratio-dependent functional response $\frac{ax(t)y(t)}{bx(t)+cy(t)}$ is selected here in the predator-prey models and the following subsystem can be obtained:

$$\left\{ \begin{array}{c} \frac{dx\left(t_{1}\right)}{dt_{1}}=rx(t_{1})(1-\frac{x(t_{1})}{K})-\frac{ax(t_{1})y(t_{1})}{bx(t_{1})+cy(t_{1})}\\ \frac{dy\left(t_{1}\right)}{dt_{1}}=-Dy(t_{1})+\frac{ex(t_{1})y(t_{1})}{bx(t_{1})+cy(t_{1})} \end{array}\right.$$ (2)

In the paper, we develop the Filippov predator-prey model by combining the above two subsystems:

$$\left\{ \begin{array}{cc} \frac{dx\left(t_{1}\right)}{dt_{1}}=rx(t_{1})(1-\frac{x(t_{1})}{K})-\frac{ax(t_{1})y(t_{1})}{1+bx(t_{1})} & x(t_{1})>ET\\ \frac{dy\left(t_{1}\right)}{dt_{1}}=-Dy(t_{1})+\frac{ex(t_{1})y(t_{1})}{1+bx(t_{1})}\\ \frac{dx\left(t_{1}\right)}{dt_{1}}=rx(t_{1})(1-\frac{x(t_{1})}{K})-\frac{ax(t_{1})y(t_{1})}{bx(t_{1})+cy(t_{1})} & x(t_{1})<ET\\ \frac{dy\left(t_{1}\right)}{dt_{1}}=-Dy(t_{1})+\frac{ex(t_{1})y(t_{1})}{bx(t_{1})+cy(t_{1})} \end{array}\right.$$ (3)

that is:

$$\left\{ \begin{array}{c} \frac{dx\left(t_{1}\right)}{dt_{1}}=rx(t_{1})(1-\frac{x(t_{1})}{K})-\frac{ax(t_{1})y(t_{1})}{\eta +bx(t_{1})+c\epsilon y(t_{1})}\\ \frac{dy\left(t_{1}\right)}{dt_{1}}=-Dy(t_{1})+\frac{ex(t_{1})y(t_{1})}{\eta +bx(t_{1})+c\epsilon y(t_{1})} \end{array}\right.$$ (4)

in which


$$\eta =\left\{ \begin{array}{cc} 1 & x(t_{1})>ET\\ 0 & x(t_{1})<ET \end{array}\right.$$

and


$$\epsilon =\left\{ \begin{array}{cc} 0 & x(t_{1})>ET\\ 1 & x(t_{1})<ET \end{array}\right.$$

Note that the ET is set by certain threshold strategy. In order to simplify the system in (4), the parameters and variables can be defined as follows:


$$t=rt_{1},x_{1}=\frac{x}{K},y_{1}=\frac{y}{K},a_{1}=\frac{aK}{r},b_{1}=bK,D_{1}=\frac{D}{K},e_{1}=\frac{eK}{r},c_{1}=cK$$

Then we can obtain:

$$\left\{ \begin{array}{c} \frac{dx_{1}\left(t\right)}{dt}=x_{1}(t)(1-x_{1}(t))-\frac{a_{1}x_{1}(t)y_{1}(t)}{\eta +b_{1}x_{1}(t)+c_{1}\epsilon y_{1}(t)}\\ \frac{dy_{1}\left(t\right)}{dt}=-D_{1}y_{1}(t)+\frac{e_{1}x_{1}(t)y_{1}(t)}{\eta +b_{1}x_{1}(t)+c_{1}\epsilon y_{1}(t)} \end{array}\right.$$ (5)

in which


$$\eta =\left\{ \begin{array}{cc} 1 & x_{1}(t)>ET\\ 0 & x_{1}(t)<ET \end{array}\right.$$

and


$$\epsilon =\left\{ \begin{array}{cc} 0 & x_{1}(t)>ET\\ 1 & x_{1}(t)<ET \end{array}\right.$$

The aim of this study is to investigate the complex dynamics including bistabilities of the ecosystem when the relative populations of the prey and predator is substantially different, by modelling the non-smooth Filippov system with multiple switchable functional responses for the very first time, which highlights the significant role of the threshold in the process of pest controls: it is seen from this work that different types of equilibrium points can occur dependent on the choice of the economic threshold (ET). Finally, the resulting conclusion is given the corresponding biological explanation. The organization of this paper is outlined as follows: Sect 2 gives a summary of how various regimes such as the sliding region, crossing region and escaping region are defined, subsequently the five different kinds of equilibria that will be discussed in the following sections of the paper will be briefly introduced. In Sect 3, the dynamical behaviors of two subsystems (i.e. the system (1) and (2) as set out in the above paragraphs) and their dynamic behaviors on the discontinuity boundary Σ (see text in Sect 2) are derived. Subsequently their equilibria together with the existence of three regimes such as the sliding, escaping and crossing domains, are derived. The dynamics of the sliding mode and various forms of equilibria within the Filippov system (i.e. the system (5) in the above paragraph), and their global asymptotic stability are discussed in Sect 4. In Sect 5, we probe the bifurcation set of equilibria and their global stability of equilibria through numerical simulations. Subsequently the paper is concluded in Sect 6 and the theoretical results are discussed in the context of biological factors and practical viewpoints.

## 2 Preliminaries

Denote


$$D=\{(x_{1},y_{1})\in R^{2+}|x_{1}\;>\;0,y_{1}\;>\;0\},\;H(Z)=H(x_{1},y_{1})=x_{1}-ET,\;F_{1}(x_{1},y_{1})=[x_{1}(t)(1-x_{1}(t))-\frac{a_{1}x_{1}(t)y_{1}(t)}{1+b_{1}x_{1}(t)},-D_{1}y_{1}(t)+\frac{e_{1}x_{1}(t)y_{1}(t)}{1+b_{1}x_{1}(t)}{]}^{T}\;=[F_{11},F_{12}{]}^{T},\;F_{2}(x_{1},y_{1})=[x_{1}(t)(1-x_{1}(t))-\frac{a_{1}x_{1}(t)y_{1}(t)}{b_{1}x_{1}(t)+c_{1}y_{1}(t)},-D_{1}y_{1}(t)+\frac{e_{1}x_{1}(t)y_{1}(t)}{b_{1}x_{1}(t)+c_{1}y_{1}(t)}{]}^{T}\;=[F_{21},F_{22}{]}^{T},\;F_{i}H=<F_{i},\nabla H>(i=1,2),\;Z=(x_{1},y_{1}).$$

Therefore, the system (5) can be rewritten as the following generalized system with discontinuous in the right-hand side as shown below:

$$\dot{Z}(t)=\left\{ \begin{array}{cc} F_{1}(Z) & Z\in G_{1},\\ F_{2}(Z) & Z\in G_{2}, \end{array}\right.$$ (6)

in which


$$G_{1}=\{H(Z)\;>\;0\},G_{2}=\{H(Z)<0\}.$$

And the discontinuous boundary between the region *G*_1_ and *G*_2_ is defined as Σ={Z∈D|H(Z)=0}, so we have $D=G_{1}⋃\Sigma ⋃G_{2}$. The discontinuous boundary Σ can be classified as the following three different regions:

(i) The Sliding region $\Sigma_{s}=\{Z\in \Sigma ,F_{1}H<0$ and $F_{2}H\;>\;0\}$, which implies that once the trajectories of the system touch the boundary $\Sigma_{s}$, it will stay in the same region.

(ii) The Crossing region $\Sigma_{c}=\{Z\in \Sigma ,F_{1}H\cdot F_{2}H\;>\;0\}$, which implies that once the trajectories of the system touch the boundary $\Sigma_{c}$, it will propagate to another region.

(iii) The Escaping region $\Sigma_{e}=\{Z\in \Sigma ,F_{1}H\;>\;0$ and *F*_2_*H*<0}, which implies that once the trajectories of the system touch the boundary $\Sigma_{e}$, it will propagate towards either region *G*_1_ or *G*_2_.

**Definition 2.1.** *[8] Provided* $F_{1}(E_{R})=0$ *and* $E_{R}\in G_{1}$*(*$F_{2}(E_{R})=0$ *and* $E_{R}\in G_{2}$*), then E*_*R*_ *is a real equilibrium of system (*6*).*

**Definition 2.2.** *[8] Provided* $F_{1}(E_{V})=0$ *and* $E_{V}\in G_{2}$*(*$F_{2}(E_{V})=0$ *and* $E_{V}\in G_{1}$*), then* $E_{V}$ *is a virtual equilibrium of system (*6*).*

**Definition 2.3.** *[8] Provided* $E_{p}\in \Sigma$ *and* $\lambda F_{1}(E_{p})+(1-\lambda )F_{2}(E_{p})=0$ , *where* 0<λ<1,*and* $\lambda =\frac{F_{2}H}{F_{2}H-F_{1}H}$, *then E*_*p*_ *is a pseudo-equilibrium of system (*6*).*

**Definition 2.4.** *[8] Provided* $F_{i}(E_{b})=0(i=1,2)$ *and* $E_{b}\in \Sigma$, *then E*_*b*_ *is a boundary equilibrium of system (*6*).*

**Definition 2.5.** *[8] Provided* $F_{i}H(E_{T})=0(i=1,2)$ *and* $E_{T}\in \Sigma$, *then E*_*T*_ *is the tangency point of system (*6*).*

**Definition 2.6.** *[8] Provided* $E_{T}\in \Sigma$, $F_{1}H(E_{T})=0$ *and* $F_{1}^{2}H(E_{T})<0$ (*or* $F_{1}^{2}H(E_{T})\;>\;0$*), then E*_*T*_ *is an invisible (or visible) tangency equilibrium of subsystem (*1*). Similarly, provided* $E_{T}\in \Sigma$, $F_{2}H(E_{T})=0$ *and* $F_{2}^{2}H(E_{T})\;>\;0$ (*or* $F_{2}^{2}H(E_{T})<0$*), then E*_*T*_ *is an invisible (or visible) tangency equilibrium of subsystem (*2*).*

## 3 Qualitative analysis of the subsystem (1) and (2)

**Lemma 3.1.** *Suppose that* $Z(t)=(x_{1}(t),y_{1}(t))$ *is any solution of system (*5*) with the initial value* $Z(t_{0})=(x_{1}(t_{0}),y_{1}(t_{0}))$, $x_{1}(t_{0})\;>\;0,y_{1}(t_{0})\;>\;0$, *then Z(t) > 0, namely* $x_{1}(t)\;>\;0,y_{1}(t)\;>\;0$.

*Proof*: As


$$\frac{dx_{1}\left(t\right)}{dt}{|}_{x_{1}=0}=[x_{1}(t)(1-x_{1}(t))-\frac{a_{1}x_{1}(t)y_{1}(t)}{\eta +b_{1}x_{1}(t)+c_{1}\epsilon y_{1}(t)}]{|}_{x_{1}=0}=0,\;\frac{dy_{1}\left(t\right)}{dt}{|}_{y_{1}=0}=[-D_{1}y_{1}(t)+\frac{e_{1}x_{1}(t)y_{1}(t)}{\eta +b_{1}x_{1}(t)+c_{1}\epsilon y_{1}(t)}]{|}_{y_{1}=0}=0.$$

Thus *Z*(*t*) > 0 as long as the initial value satisfies $x_{1}(t_{0})\;>\;0,y_{1}(t_{0}))\;>\;0$. □

**Lemma 3.2.** *Suppose* $Z(t)=(x_{1}(t),y_{1}(t))$ *to be the solution of system (*5*), then the set* $\Omega =\{(x_{1},y_{1})\in R^{2+}|x_{1}\leq 1,\frac{e_{1}}{a_{1}}x_{1}+y_{1}\leq \frac{M}{\lambda }\}$ *is positively invariant and attracting for any given initial values in R*^2 + ^.

*Proof*: It follows that


$$\frac{dx_{1}\left(t\right)}{dt}=x_{1}(t)(1-x_{1}(t))-\frac{a_{1}x_{1}(t)y_{1}(t)}{\eta +b_{1}x_{1}(t)+c_{1}\epsilon y_{1}(t)}\leq x_{1}(t)(1-x_{1}(t)).$$

By solving the above equation, we can obtain that:


$$x_{1}(t)\leq \frac{1}{1+ce^{-t}}(c\geq 0),$$

which results in


$$\lim_{t\to \infty }x_{1}(t)\leq 1.$$

Then we can get


$$x_{1}(t)\leq 1.$$

Define the function $W\left(t,x\right)=\frac{e_{1}}{a_{1}}x_{1}\left(t\right)+y_{1}\left(t\right)$ , then:


$$\frac{dW\left(t,x\right)}{dt}=\frac{e_{1}}{a_{1}}\frac{\text{d}x_{1}\left(t\right)}{\text{d}t}+\frac{\text{d}y_{1}\left(t\right)}{\text{d}t}=\frac{e_{1}}{a_{1}}x_{1}(t)(1-x_{1}(t))-D_{1}y_{1}\left(t\right),$$

which is the upper right derivative of W(t,x) along a solution of the system in (5) with respected to time and for $0<\lambda \leq D_{1},$ we have


$$\begin{array}{c} \frac{dW\left(t,x\right)}{dt}+\lambda W\left(t,x\right)=\frac{e_{1}}{a_{1}}x_{1}(t)(1+\lambda -x_{1}(t))+\left(\lambda -D_{1}\right)y_{1}\left(t\right)\\ \leq \frac{e_{1}}{a_{1}}(\lambda +1)x_{1}(t)-\frac{e_{1}x_{1}(t{)}^{2}}{a_{1}}=-\frac{e_{1}}{a_{1}}[x_{1}(t{)}^{2}-(\lambda +1)x_{1}(t)]\\ =-\frac{e_{1}}{a_{1}}[x_{1}(t)-\frac{\left(\lambda +1\right)}{2}{]}^{2}+\frac{(\lambda +1{)}^{2}e_{1}}{4a_{1}}\leq \frac{(\lambda +1{)}^{2}e_{1}}{4a_{1}}. \end{array}$$

Thus there exists a positive constant number $M=\frac{{\left(\lambda +1\right)}^{2}e_{1}}{4a_{1}}$, such that


$$\frac{dW\left(t,x\right)}{dt}+\lambda W\left(t,x\right)\leq M,$$

By solving the above equation it produces:


$$W\left(t,x\right)=e^{-\lambda t}[\int Me^{\lambda t}dx+C]=e^{-\lambda t}[C+\frac{Me^{\lambda t}}{\lambda }]=\frac{M}{\lambda }+Ce^{-\lambda t}\to \frac{M}{\lambda }(t\to \infty )$$

Hence W(t,x) is ultimately bounded by a constant, namely $\frac{e_{1}}{a_{1}}x_{1}+y_{1}\leq \frac{M}{\lambda }$, thus Ω is positively invariant and attracting for any given initial values in *R*^2 + ^. □

### 3.1 Dynamical behaviors of the subsystem (1)

When *x*_1_(*t*)>*ET* the Filippov system (6) in section 2 is qualitatively dependent on the subsystem (1). There are three equilibrium points in the subsystem(1) namely: *E*_01_ = (0,0), *E*_11_ = (1,0), $E_{1}=(x_{1}^{*},y_{1}^{*}),$ in which


$$x_{1}^{*}=\frac{D_{1}}{e_{1}-b_{1}D_{1}},y_{1}^{*}=\frac{\left(1-x_{1}^{*})(1+b_{1}x_{1}^{*}\right)}{a_{1}}.$$

As $(x_{1}^{*},y_{1}^{*})\in R^{2+}$, namely $x_{1}^{*}\;>\;0,e_{1}-b_{1}D_{1}\;>\;0$, we can obtain that $D_{1}<\frac{e_{1}}{b_{1}+1};$ $y_{1}^{*}\;>\;0,1-x_{1}^{*}\;>\;0$, we can obtain that $\frac{e_{1}}{b_{1}+1}<\frac{e_{1}}{b_{1}},$ so we can get that $D_{1}<\frac{e_{1}}{b_{1}+1}<\frac{e_{1}}{b_{1}}$.

**Theorem 3.3.** (*i) The equilibrium E*_01_ = (0,0) *is in a saddle and it is unstable all the time. (ii) The equilibrium point E*_11_ = (1,0) *is in a saddle when* $D_{1}<\frac{e_{1}}{b_{1}+1}$, *and it is locally asymptotically stable when* $D_{1}>\frac{e_{1}}{b_{1}+1}$. (*iii) The interior equilibrium point* $E_{1}=(x_{1}^{*},y_{1}^{*})$ *is locally asymptotically stable when* $\frac{e_{1}(b_{1}-1)}{b_{1}(1+b_{1})}<D_{1}<\frac{e_{1}}{b_{1}+1}$.

*Proof*: Consider the Jacobian matrix about the equilibrium point of the subsystem (1):


$$J=(\begin{array}{c} \begin{array}{cc} 1-2x_{1}-\frac{a_{1}y_{1}}{(1+b_{1}x_{1}{)}^{2}} & -\frac{a_{1}x_{1}}{1+b_{1}x_{1}}\\ \frac{e_{1}y_{1}}{(1+b_{1}x_{1}{)}^{2}} & -D_{1}+\frac{e_{1}x_{1}}{1+b_{1}x_{1}} \end{array} \end{array}).$$

(i) Let $x_{1}=y_{1}=0$, we can get the $J(E_{01})=(\begin{array}{c} \begin{array}{cc} 1 & 0\\ 0 & -D_{1} \end{array} \end{array}).$ Then we obtain the eigenvalues of *J*(*E*_01_) will be $\lambda_{1}=1,\lambda_{2}=-D_{1}<0$, so the equilibrium *E*_01_ = (0,0) is saddle and unstable all the time.

(ii) Let $x_{1}=1,y_{1}=0$, we can get the $J(E_{11})=(\begin{array}{c} \begin{array}{cc} -1 & -\frac{a_{1}}{1+b_{1}}\\ 0 & -D_{1}+\frac{e_{1}}{1+b_{1}} \end{array} \end{array}),$ then we obtain the eigenvalues of *J*(*E*_11_) is $\lambda_{1}=-1,\lambda_{2}=-D_{1}\;+\;\frac{e_{1}}{1+b_{1}}$. When $D_{1}<\frac{e_{1}}{b_{1}+1}$, then $\lambda_{2}\;>\;0$, so *E*_11_ = (1,0) is saddle. Similarly, when $D_{1}>\frac{e_{1}}{b_{1}+1}$, then $\lambda_{2}<0$, so *E*_11_ = (1,0) is locally asymptotically stable.

(iii) Let $x_{1}=x_{1}^{*},y_{1}=y_{1}^{*}$, we can get the


$$J(E_{1})=(\begin{array}{c} \begin{array}{cc} 1-2x_{1}^{*}-\frac{1-x_{1}^{*}}{1+b_{1}x_{1}^{*}} & -\frac{a_{1}x_{1}^{*}}{1+b_{1}x_{1}^{*}}\\ \frac{e_{1}(1-x_{1}^{*})}{a_{1}(1+b_{1}x_{1}^{*})} & -D_{1}+\frac{e_{1}x_{1}^{*}}{1+b_{1}x_{1}^{*}} \end{array} \end{array}),$$

then we obtain the result that all eigenvalues of *J*(*E*_1_) are negative when *tr*(*J*(*E*_1_))<0 and $|J(E_{1})|\;>\;0$, that is, $\frac{e_{1}(b_{1}-1)}{b_{1}(1+b_{1})}<D_{1}<\frac{e_{1}}{b_{1}+1}$, the interior point $E_{1}=(x_{1}^{*},y_{1}^{*})$ is locally asymptotic stable. □

**Theorem 3.4.** *The interior point* $E_{1}=(x_{1}^{*},y_{1}^{*})$ *is globally asymptotic stable when* $b(1\;-\;\frac{D_{1}}{e_{1}-b_{1}D_{1}})<1$.

*Proof*: Define


$$V_{1}=D_{1}[x_{1}-x_{1}^{*}-x_{1}^{*}ln\frac{x_{1}}{x_{1}^{*}}]+D_{2}[y_{1}-y_{1}^{*}-y_{1}^{*}ln\frac{y_{1}}{y_{1}^{*}}]$$

where *D*_1_ and *D*_2_ are arbitrary positive constants. Then we can obtain:

$$\frac{dV_{1}}{dt}=D_{1}\frac{\left(x_{1}-x_{1}^{*}\right)}{x_{1}}\frac{dx_{1}}{dt}+D_{2}\frac{\left(y_{1}-y_{1}^{*}\right)}{y_{1}}\frac{dy_{1}}{dt}$$ (7)

Substituting the subsystem (1) into the above equation (7) which generates


$$\frac{dV_{1}}{dt}=D_{1}(x_{1}-x_{1}^{*}{)}^{2}[-1+\frac{a_{1}b_{1}y_{1}^{*}}{\left(1+b_{1}x_{1})(1+b_{1}x_{1}^{*}\right)}]\;+\frac{\left(x_{1}-x_{1}^{*})(y_{1}-y_{1}^{*}\right)}{\left(1+b_{1}x_{1})(1+b_{1}x_{1}^{*}\right)}e_{1}D_{2}-a_{1}D_{1}(1+b_{1}x_{1}^{*}).$$

Put *D*_1_ = 1 and $D_{2}=\frac{a_{1}(1+b_{1}x_{1}^{*})}{e_{1}}$, then


$$\frac{dV_{1}}{dt}=(x_{1}-x_{1}^{*}{)}^{2}[-1+\frac{a_{1}b_{1}y_{1}^{*}}{\left(1+b_{1}x_{1})(1+b_{1}x_{1}^{*}\right)}]\;<(x_{1}-x_{1}^{*}{)}^{2}[-1+\frac{a_{1}b_{1}y_{1}^{*}}{\left(1+b_{1}x_{1}^{*}\right)}].$$

When $-1\;+\;\frac{a_{1}b_{1}y_{1}^{*}}{\left(1+b_{1}x_{1}^{*}\right)}<0$, then it has that $\frac{dV_{1}}{dt}<0$. By employing the value of $y_{1}^{*}$, we can get $b(1-x_{1}^{*})<1$, namely $b(1-\frac{D_{1}}{e_{1}-b_{1}D_{1}})<1$. □

### 3.2 Dynamical behaviors of the subsystem (2)

When *x*_1_(*t*)<*ET*, the Filippov system (6) is qualitatively dependent on the subsystem (2). There are three equilibrium points in the subsystem (1): *E*_20_ = (0,0), *E*_21_ = (1,0), $E_{2}=(x_{2}^{*},y_{2}^{*}),$ in which


$$x_{2}^{*}=1+\frac{a_{1}D_{1}b_{1}-a_{1}e_{1}}{c_{1}e_{1}},y_{2}^{*}=\frac{(1-x_{2}^{*})e_{1}x_{2}^{*}}{a_{1}D_{1}}.$$

As $(x_{2}^{*},y_{2}^{*})\in R^{2+}$, namely $x_{2}^{*}\;>\;0$, we can obtain that $D_{1}<\frac{e_{1}}{b_{1}};$ $y_{2}^{*}\;>\;0,1-x_{2}^{*}\;>\;0$, we can obtain that $\frac{a_{1}e_{1}-c_{1}e_{1}}{a_{1}b_{1}}<D_{1}$, so we can get that $\frac{a_{1}e_{1}-c_{1}e_{1}}{a_{1}b_{1}}<D_{1}<\frac{e_{1}}{b_{1}}$.

**Theorem 3.5.** (*i) The equilibrium E*_20_ = (0,0) *is in a saddle and it is unstable all the time. (ii) The equilibrium point E*_21_ = (1,0) *is also in a saddle when* $D_{1}<\frac{e_{1}}{b_{1}}$, *and it is locally asymptotically stable when* $D_{1}>\frac{e_{1}}{b_{1}}$. (*iii) The interior point* $E_{2}=(x_{2}^{*},y_{2}^{*})$ *is locally asymptotically stable when* $D_{1}<\frac{e_{1}}{b_{1}+1}$ *and* $a_{1}<\frac{e_{1}D_{1}+\frac{e_{1}^{2}}{e_{1}-D_{1}}}{e_{1}+D_{1}}$.

*Proof*: Consider the Jacobian matrix about the equilibrium point of the subsystem (2):


$$J=(\begin{array}{c} \begin{array}{cc} 1-2x_{1}-\frac{a_{1}c_{1}y_{1}^{2}}{(b_{1}x_{1}+c_{1}y_{1}{)}^{2}} & -\frac{a_{1}b_{1}x_{1}^{2}}{(b_{1}x_{1}+c_{1}y_{1}{)}^{2}}\\ \frac{e_{1}c_{1}y_{1}^{2}}{(b_{1}x_{1}+c_{1}y_{1}{)}^{2}} & -D_{1}+\frac{e_{1}b_{1}x_{1}^{2}}{(b_{1}x_{1}+c_{1}y_{1}{)}^{2}} \end{array} \end{array}).$$

(i) Let $x_{1}=y_{1}=0$, we can get the $J(E_{20})=(\begin{array}{c} \begin{array}{cc} 1 & 0\\ 0 & -D_{1} \end{array} \end{array}).$ Then we obtain the eigenvalues of *J*(*E*_20_) will be $\lambda_{1}=1,\lambda_{2}=-D_{1}<0$, therefore the equilibrium *E*_20_ = (0,0) is in a saddle and it is unstable all the time.

(ii) Let $x_{1}=1,y_{1}=0$, we can get the $J(E_{21})=(\begin{array}{c} \begin{array}{cc} -1 & -\frac{a_{1}}{b_{1}}\\ 0 & -D_{1}+\frac{e_{1}}{b_{1}} \end{array} \end{array}),$ then we obtain the eigenvalues of *J*(*E*_21_) will be $\lambda_{1}=-1,\lambda_{2}=-D_{1}+\frac{e_{1}}{b_{1}}$. When $D_{1}<\frac{e_{1}}{b_{1}}$, then $\lambda_{2}\;>\;0$, so *E*_21_ = (1,0) is in a saddle. Similarly, when $D_{1}>\frac{e_{1}}{b_{1}}$, then $\lambda_{2}<0$, so *E*_21_ = (1,0) is locally asymptotically stable.

(iii) Let $x_{1}=x_{2}^{*},y_{1}=y_{2}^{*}$, we can get the


$$J(E_{2})=(\begin{array}{c} \begin{array}{cc} 1-2x_{2}^{*}-\frac{a_{1}c_{1}y_{2}^{{*}^{2}}}{(b_{1}x_{2}^{*}+c_{1}y_{2}^{*}{)}^{2}} & -\frac{a_{1}b_{1}x_{2}^{{*}^{2}}}{(b_{1}x_{2}^{*}+c_{1}y_{2}^{*}{)}^{2}}\\ \frac{e_{1}c_{1}y_{2}^{{*}^{2}}}{(b_{1}x_{2}^{*}+c_{1}y_{2}^{*}{)}^{2}} & -D_{1}+\frac{e_{1}b_{1}x_{2}^{{*}^{2}}}{(b_{1}x_{2}^{*}+c_{1}y_{2}^{*}{)}^{2}} \end{array} \end{array}),$$

then we obtain the result that all eigenvalues of *J*(*E*_2_) are negative when *tr*(*J*(*E*_2_))<0 and $|J(E_{2})|\;>\;0$, that is, $D_{1}<\frac{e_{1}}{b_{1}+1}$ and $a_{1}<\frac{e_{1}D_{1}+\frac{e_{1}^{2}}{e_{1}-D_{1}}}{e_{1}+D_{1}}$, the interior point $E_{2}=(x_{2}^{*},y_{2}^{*})$ is locally asymptotically stable. □

**Theorem 3.6.** *The interior point* $E_{2}=(x_{2}^{*},y_{2}^{*})$ *is globally asymptotically stable when* $a_{1}b_{1}\;-\;e_{1}c_{1}<0$.

*Proof*: We select the Dulac fuction $G(x,y)=\frac{1}{x_{1}y_{1}}$, then the following can be obtained:


$$\frac{\partial (GF_{21})}{\partial x}+\frac{\partial (GF_{22})}{\partial y}=-\frac{1}{y_{1}}+\frac{a_{1}b_{1}-e_{1}c_{1}}{(b_{1}x_{1}+c_{1}y_{1}{)}^{2}}.$$

Thus based on the Bendixson-Dulac criterion, when $a_{1}b_{1}\;-\;e_{1}c_{1}<0$ there are no any closed orbits in the region *G*_2_, therefore the interior point $E_{2}=(x_{2}^{*},y_{2}^{*})$ is globally asymptotic stable. □

## 4 The dynamic behaviors on ΣSigma and equilibria

This section will be divided into three parts, firstly the existence of the sliding domain, escaping domain and crossing domain will be studied. Then the sliding mode dynamics will be focused and finally different kinds of equilibria points in Filippov system (6) will be examined in more details.

### 4.1 The existence of the sliding domain, escaping domain and crossing domain

Let’s consider the discontinuous boundary Σ and through simple evaluation on the boundary gives:


$$F_{1}H=x_{1}(t)(1-x_{1}(t))-\frac{a_{1}x_{1}(t)y_{1}(t)}{1+b_{1}x_{1}(t)}=0,\;F_{2}H=x_{1}(t)(1-x_{1}(t))-\frac{a_{1}x_{1}(t)y_{1}(t)}{b_{1}x_{1}(t)+c_{1}y_{1}(t)}=0,$$

where ∇H=(1,0) and *x*_1_(*t*) = *ET*. Hence, we can get that:


$$y_{s1}=\frac{\left(1-ET)(1+b_{1}ET\right)}{a_{1}}\;y_{s2}=\frac{(1-ET)b_{1}ET}{a_{1}-c_{1}(1-ET)}$$

Next, consider the following three cases:

**case 1:** When $a_{1}-c_{1}(1-ET)-b_{1}c_{1}ET(1-ET)\;>\;0$, then $a_{1}-c_{1}(1-ET)\;>\;0$, thus $y_{s2}\;>\;0$ and $y_{s1}>y_{s2}$, we can obtain that:

(i) Sliding region $\Sigma_{s}=\{(x_{1},y_{1})\in \Sigma \;|\;y_{s2}<y_{1}<y_{s1}\}$.

(ii) Crossing region $\Sigma_{c}=\{(x_{1},y_{1})\in \Sigma \;|\;0<y_{1}<y_{s2}\}⋃\{(x_{1},y_{1})\in \Sigma \;|\;y_{1}>y_{s1}\}.$

**case 2:** When $a_{1}-c_{1}(1-ET)\;>\;0$ and $a_{1}-c_{1}(1-ET)-b_{1}c_{1}ET(1-ET)<0$, thus $y_{s2}\;>\;0$ and $y_{s1}<y_{s2}$, we can obtain that:

(i) Sliding region $\Sigma_{s}=\{(x_{1},y_{1})\in \Sigma \;|\;y_{s1}<y_{1}<y_{s2}\}$.

(ii) Crossing region $\Sigma_{c}=\{(x_{1},y_{1})\in \Sigma \;|\;0<y_{1}<y_{s1}\}⋃\{(x_{1},y_{1})\in \Sigma \;|\;y_{1}>y_{s2}\}.$

**case 3:** If $a_{1}-c_{1}(1-ET)<0$, then $a_{1}-c_{1}(1-ET)-b_{1}c_{1}ET(1-ET)<0$, thus *y*_*s*2_<0 and $y_{s1}>y_{s2}$, we can get that:

(i) Escaping region $\Sigma_{s}=\{(x_{1},y_{1})\in \Sigma \;|\;0<y_{1}<y_{s1}\}$.

(ii) Crossing region $\Sigma_{s}=\{(x_{1},y_{1})\in \Sigma \;|\;y_{1}>y_{s1}\}$.

According to the above results the following theorem can be produced:

**Theorem 4.1.** *The sliding region and escaping region can not exist at the same time.*

### 4.2 Sliding mode dynamics

By using the Utkin’s equivalent control method the following can be obtained:


$$\frac{dH}{dt}=\frac{dx_{1}(t)}{dt}=x_{1}(t)(1-x_{1}(t))-\frac{a_{1}x_{1}(t)y_{1}(t)}{\eta +b_{1}x_{1}(t)+c_{1}\epsilon y_{1}(t)}=0.$$

When *x*_1_(*t*) = *ET* and by solving the above equation:


$$\eta +b_{1}x_{1}(t)+c_{1}\epsilon y_{1}(t)=\frac{a_{1}x_{1}(t)y_{1}(t)}{x_{1}(t)(1-x_{1}(t))},$$

then we can get:


$$\frac{dy_{1}\left(t\right)}{dt}=-D_{1}y_{1}(t)+\frac{e_{1}x_{1}(t)y_{1}(t)}{\eta +b_{1}x_{1}(t)+c_{1}\epsilon y_{1}(t)}\;=-D_{1}y_{1}(t)+\frac{e_{1}}{a_{1}}(x_{1}-x_{1}^{2})\;=-D_{1}y_{1}(t)+\frac{e_{1}ET(1-ET)}{a_{1}}≜\phi (y_{1}).$$

### 4.3 Five kinds of equilibriums of Filippov system (6)

In this section five different kinds of equilibriums in the Filippov system (6) is discussed here. It follows from the section 3 that $E_{1}=(\frac{D_{1}}{e_{1}-b_{1}D_{1}},\frac{\left(1-x_{1}^{*})(1+b_{1}x_{1}^{*}\right)}{a_{1}})$ is the unique positive equilibrium of the subsystem (1), and $E_{2}=(1\;+\;\frac{a_{1}D_{1}b_{1}-a_{1}e_{1}}{c_{1}e_{1}},\frac{(1-x_{2}^{*})e_{1}x_{2}^{*}}{a_{1}D_{1}})$ is the unique positive equilibrium of the subsystem (2). The nature and types of the above two equilibriums can be studied as follow:

(i) When the condition that $\frac{D_{1}}{e_{1}-b_{1}D_{1}}>ET$ is satisfied, then *E*_1_ is in a real equilibrium and hereby it is termed as $E_{R}^{1}$. Otherwise *E*_1_ is a virtual equilibrium and hereby termed as $E_{V}^{1}$.

(ii) When the condition that $1+\frac{a_{1}D_{1}b_{1}-a_{1}e_{1}}{c_{1}e_{1}}<ET$ is satisfied, then *E*_2_ is in a real equilibrium which hereby termed as $E_{R}^{2}$. If not, then *E*_2_ is in a virtual equilibrium and hereby termed as $E_{V}^{2}$.

**Pseudo-equilibrium:** There are two different ways to satisfy the pseudo-equilibrium condition $E_{p}(ET,y_{p})\in \Sigma$ as it is to be shown in the following: one way is to obtain the condition by solving the equation $\phi (y_{p})=0$, where $y_{p}=\frac{e_{1}ET(1-ET)}{a_{1}D_{1}}$. The other is from the definition 2.3:


$$\lambda (\begin{array}{c} \begin{array}{c} x_{1}(1-x_{1})-\frac{a_{1}x_{1}y_{1}}{1+b_{1}x_{1}}\\ -D_{1}y_{1}+\frac{e_{1}x_{1}y_{1}}{1+b_{1}x_{1}} \end{array} \end{array})+(1-\lambda )(\begin{array}{c} \begin{array}{c} x_{1}(1-x_{1})-\frac{a_{1}x_{1}y_{1}}{b_{1}x_{1}+c_{1}y_{1}}\\ -D_{1}y_{1}+\frac{e_{1}x_{1}y_{1}}{\;b_{1}x_{1}+c_{1}y_{1}} \end{array} \end{array})=(\begin{array}{c} \begin{array}{c} 0\\ 0 \end{array} \end{array}).$$

By solving the above equations the following can be obtained:


$$\lambda =\frac{x_{1}(1-x_{1})-\frac{a_{1}x_{1}y_{1}}{b_{1}x_{1}+c_{1}y_{1}}}{\frac{a_{1}x_{1}y_{1}}{b_{1}x_{1}+c_{1}y_{1}}-\frac{a_{1}x_{1}y_{1}}{1+b_{1}x_{1}}},$$

then by substituting the value of *λ* into the above equation it yields the pseudo-equilibrium $E_{p}(ET,y_{p})$ such that:


$$y_{p}=\frac{e_{1}ET(1-ET)}{a_{1}D_{1}}.$$

According to $\phi^{\prime }(y_{1})=-D_{1}<0$ and the stability theory of the ODE it can be shown that the pseudo-equilibrium *E*_*p*_ is locally asymptotic stable.

**Theorem 4.2.** (*i) When* $a_{1}-c_{1}(1-ET)-b_{1}c_{1}ET(1-ET)\;>\;0$, *the pseudo-equilibrium E*_*p*_ *exists iff the real equilibrium* $E_{R}^{1}$ *and* $E_{R}^{2}$ *are both coexisted.*

(*ii) When* $a_{1}-c_{1}(1-ET)\;>\;0$ *and* $a_{1}-c_{1}(1-ET)-b_{1}c_{1}ET(1-ET)<0$, *the pseudo-equilibrium E*_*p*_ *exists iff the virtual equilibrium* $E_{V}^{1}$ *and* $E_{V}^{2}$ *are both coexisted.*

*Proof*: Due to the fact that:


$$y_{1}-y_{s1}=\frac{e_{1}ET(1-ET)}{a_{1}D_{1}}-\frac{\left(1-ET)(1+b_{1}ET\right)}{a_{1}}\;=\frac{\left(1-ET)(e_{1}ET-D_{1}-b_{1}ETD_{1}\right)}{a_{1}D_{1}}\;=\frac{\left(1-ET)(e_{1}-D_{1}b_{1})(ET-\frac{D_{1}}{e_{1}-b_{1}D_{1}}\right)}{a_{1}D_{1}},\;y_{1}-y_{s2}=\frac{e_{1}ET(1-ET)}{a_{1}D_{1}}-\frac{(1-ET)b_{1}ET}{a_{1}-c_{1}(1-ET)}\;=\frac{ET(1-ET)[a_{1}e_{1}-e_{1}c_{1}(1-ET)-a_{1}D_{1}b_{1}]}{a_{1}D_{1}[a_{1}-c_{1}(1-ET)]}\;=\frac{ET(1-ET)}{a_{1}D_{1}[a_{1}-c_{1}(1-ET)]}\cdot \frac{ET-1-\frac{a_{1}D_{1}b_{1}-a_{1}e_{1}}{c_{1}e_{1}}}{e_{1}c_{1}},$$

where $x_{1}^{*}=\frac{D_{1}}{e_{1}-b_{1}D_{1}}$, $x_{2}^{*}=1+\frac{a_{1}D_{1}b_{1}-a_{1}e_{1}}{c_{1}e_{1}}$, $1-ET\;>\;0,e_{1}-D_{1}b_{1}\;>\;0$.

Thus (i) When $a_{1}-c_{1}(1-ET)-b_{1}c_{1}ET(1-ET)\;>\;0$, then the pseudo-equilibrium *E*_*p*_ exists $\Leftrightarrow y_{s2}<y_{1}<y_{s1}\Leftrightarrow x_{2}^{*}<ET<x_{1}^{*}\Leftrightarrow$ and the real equilibrium $E_{R}^{1}$ and $E_{R}^{2}$ are both coexisted.

(ii) When $a_{1}-c_{1}(1-ET)\;>\;0$ and $a_{1}-c_{1}(1-ET)-b_{1}c_{1}ET(1-ET)<0$, the pseudo-equilibrium *E*_*p*_ exists $\Leftrightarrow y_{s1}<y_{1}<y_{s2}\Leftrightarrow ET>x_{1}^{*}$ and $ET<x_{2}^{*}\Leftrightarrow$ then the virtual equilibrium $E_{V}^{1}$ and $E_{V}^{2}$ are both coexisted. □

**Boundary equilibrium:** It is from the definition 2.4 that the boundary equilibrium should respectively satisfy the following equation:

$$\left\{ \begin{array}{c} \frac{dx_{1}\left(t\right)}{dt}=x_{1}(t)(1-x_{1}(t))-\frac{a_{1}x_{1}(t)y_{1}(t)}{1+b_{1}x_{1}(t)}=0\\ \frac{dy_{1}\left(t\right)}{dt}=-D_{1}y_{1}(t)+\frac{e_{1}x_{1}(t)y_{1}(t)}{1+b_{1}x_{1}(t)}=0\\ x_{1}(t)=ET \end{array}\right.$$ (8)

and

$$\left\{ \begin{array}{c} \frac{dx_{1}\left(t\right)}{dt}=x_{1}(t)(1-x_{1}(t))-\frac{a_{1}x_{1}(t)y_{1}(t)}{b_{1}x_{1}(t)+c_{1}y_{1}(t)}=0\\ \frac{dy_{1}\left(t\right)}{dt}=-D_{1}y_{1}(t)+\frac{e_{1}x_{1}(t)y_{1}(t)}{b_{1}x_{1}(t)+c_{1}y_{1}(t)}=0\\ x_{1}(t)=ET \end{array}\right.$$ (9)

Therefore, the Eq (8) has the solution if and only if $ET=\frac{D_{1}}{e_{1}-b_{1}D_{1}}$, then the boundary equilibrium can be obtained:


$$E_{b}^{1}=(ET,\frac{\left(1-ET)(1+b_{1}ET\right)}{a_{1}}).$$

Similarly, the Eq (9) has the solution if and only if $ET=1\;+\;\frac{a_{1}D_{1}b_{1}-a_{1}e_{1}}{c_{1}e_{1}}$, then the boundary equilibrium can be obtained:


$$E_{b}^{2}=(ET,\frac{(1-ET)b_{1}ET}{a_{1}-c_{1}(1-ET)}).$$

**Tangent point:** By putting *F*_1_*H* = 0 and *F*_2_*H* = 0, then the two tangent point can be computed:


$$E_{T}^{1}=(ET,\frac{\left(1-ET)(1+b_{1}ET\right)}{a_{1}}),E_{T}^{2}=(ET,\frac{(1-ET)b_{1}ET}{a_{1}-c_{1}(1-ET)}).$$

**Theorem 4.3.** (*i) When* $D_{1}>\frac{e_{1}ET}{1+b_{1}ET}$, *then the tangent point* $E_{T}^{1}$ *is visible.*

(*ii) When* $\frac{(1-ET)b_{1}ET}{a_{1}-c_{1}(1-ET)}<\frac{(e_{1}-b_{1}D_{1})ET}{D_{1}c_{1}}$, *then the tangent point* $E_{T}^{2}$ *is visible.*

*Proof*: (i) Through calculation we can get:


$$F_{1}H\;{|}_{E_{T}^{1}}=x_{1}(t)(1-x_{1}(t))-\frac{a_{1}x_{1}(t)y_{1}(t)}{1+b_{1}x_{1}(t)}\;{|}_{E_{T}^{1}}=0,\;F_{1}^{2}H\;{|}_{E_{T}^{1}}=\frac{\partial (F_{1}H)}{\partial z}F_{1}H\;{|}_{E_{T}^{1}}\;=(\begin{array}{c} \begin{array}{c} 1-2x_{1}(t)-\frac{a_{1}y_{1}(t)}{[1+b_{1}x_{1}(t){]}^{2}}\\ -\frac{a_{1}x_{1}(t)}{1+b_{1}x_{1}(t)} \end{array} \end{array})(\begin{array}{c} \begin{array}{c} x_{1}(t)(1-x_{1}(t))-\frac{a_{1}x_{1}(t)y_{1}(t)}{1+b_{1}x_{1}(t)}\\ -D_{1}y_{1}(t)+\frac{e_{1}x_{1}(t)y_{1}(t)}{1+b_{1}x_{1}(t)} \end{array} \end{array})\;{|}_{E_{T}^{1}}\;=\frac{a_{1}D_{1}x_{1}(t)y_{1}(t)}{1+b_{1}x_{1}(t)}-\frac{e_{1}a_{1}x_{1}^{2}(t)y_{1}(t)}{[1+b_{1}x_{1}(t){]}^{2}}\;{|}_{E_{T}^{1}}\;=x_{1}(t)(1-x_{1}(t))(D_{1}-\frac{e_{1}x_{1}(t)}{1+b_{1}x_{1}(t)}).$$

Thus, $E_{T}^{1}$ is visible $⇐F_{1}^{2}H\;>\;0\Leftrightarrow D_{1}>\frac{e_{1}ET}{1+b_{1}ET}.$

(ii) Similarly we can obtain:


$$F_{2}H\;{|}_{E_{T}^{2}}=x_{1}(t)(1-x_{1}(t))-\frac{a_{1}x_{1}(t)y_{1}(t)}{b_{1}x_{1}(t)+c_{1}y_{1}(t)}\;{|}_{E_{T}^{2}}=0,\;F_{2}^{2}H\;{|}_{E_{T}^{2}}=\frac{\partial (F_{2}H)}{\partial z}F_{2}H\;{|}_{E_{T}^{2}}\;=(\begin{array}{c} \begin{array}{c} 1-2x_{1}(t)-\frac{a_{1}c_{1}y_{1}^{2}(t)}{[b_{1}x_{1}(t)+c_{1}y_{1}(t){]}^{2}}\\ -\frac{a_{1}b_{1}x_{1}^{2}(t)}{[b_{1}x_{1}(t)+c_{1}y_{1}(t){]}^{2}} \end{array} \end{array})(\begin{array}{c} \begin{array}{c} x_{1}(t)(1-x_{1}(t))-\frac{a_{1}x_{1}(t)y_{1}(t)}{b_{1}x_{1}(t)+c_{1}y_{1}(t)}\\ -D_{1}y_{1}(t)+\frac{a_{1}x_{1}(t)y_{1}(t)}{b_{1}x_{1}(t)+c_{1}y_{1}(t)} \end{array} \end{array})\;{|}_{E_{T}^{2}}\;=\frac{a_{1}b_{1}x_{1}^{2}(t)D_{1}y_{1}(t)}{[b_{1}x_{1}(t)+c_{1}y_{1}(t){]}^{2}}-\frac{e_{1}a_{1}b_{1}x_{1}^{3}(t)y_{1}(t)}{[b_{1}x_{1}(t)+c_{1}y_{1}(t){]}^{3}}\;{|}_{E_{T}^{2}}\;=\frac{b_{1}x_{1}(t)(1-x_{1}(t))}{b_{1}x_{1}(t)+c_{1}y_{1}(t)}(D_{1}-\frac{e_{1}x_{1}(t)}{b_{1}x_{1}(t)+c_{1}y_{1}(t)}).$$

Thus, $E_{T}^{2}$ is visible $⇐F_{2}^{2}H<0\Leftrightarrow \frac{(1-ET)b_{1}ET}{a_{1}-c_{1}(1-ET)}<\frac{(e_{1}-b_{1}D_{1})ET}{D_{1}c_{1}}.$ □

## 5 Numerical simulation and bifurcation analysis

In this section the bifurcation set of equilibria and the global stability of equilibria will be assessed by means of numerical simulation methods.

### 5.1 Bifurcation set of equilibria

It can be seen from the above that the dynamics of the Filippov system (5) are greatly dependent on the ET and equilibria points of the system. Moreover, the occurrence of various types of equilibrium points are also dependent on the value of ET and also the death rate of Predators *D*_1_. Thus the bifurcation diagram as function of bifurcation parameters ET and *D*_1_ is constructed in order to explore the richness of various possible dynamics of the Filippov system (5). Three critical curves are defined as follows:


$$L_{1}=\{(D_{1},ET)\;|\;ET=\frac{D_{1}}{e_{1}-b_{1}D_{1}}\}\;L_{2}=\{(D_{1},ET)\;|\;ET=1+\frac{a_{1}D_{1}b_{1}-a_{1}e_{1}}{e_{1}c_{1}}\}\;L_{3}=\{(D_{1},ET)\;|\;D_{1}=\frac{e_{1}}{b_{1}+1}\}$$

By putting the $y_{p}=y_{s1}$ and $y_{p}=y_{s2}$, then we can obtain the curves *L*_1_ and *L*_2_ can be plotted respectively to study the relationship between pseudo-equilibrium *E*_*p*_ and the sliding segment $\Sigma_{s}$, namely $y_{s2}<y_{1}<y_{s1}$ or $y_{s1}<y_{1}<y_{s2}$. Also on the left of the curve *L*_1_ it depicts the interior equilibrium point *E*_1_ which is labelled as $E_{1}^{V}$, and it turns into $E_{1}^{R}$ on the right. Similarly, the curve *L*_2_ is the dividing line between $E_{2}^{V}$ and $E_{2}^{R}$. Also, the curve *L*_3_ is the dividing line for the existence of the interior equilibrium point.

**Fig 1:**
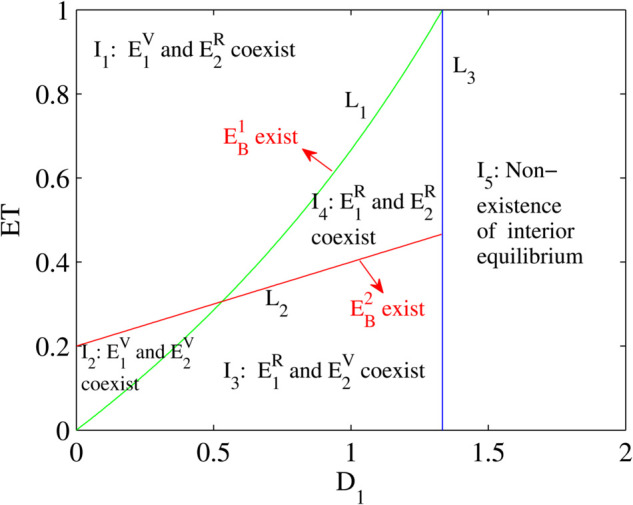
Bifurcation set of Filippov system (5)’s equilibria, where the values of other fixed parameters are $a_{1}=0.4,b_{1}=0.5,c_{1}=0.5,e_{1}=2$.

By fixing all other parameters $a_{1}=0.4,b_{1}=0.5,c_{1}=0.5,e_{1}=2$, then the $D_{1}\;-\;ET$ parameter plane (Fig 1) can be divided into five regions by these three curves, and the existence of possible equilibria is indicated in each area. It is seen that the pseudo-equilibrium *E*_*p*_ coexists in the region *I*_2_ and *I*_4_. It is worth mentioning that the boundary equilibria is located only along the curves. Moreover, if the parameter *D*_1_ is fixed to 1, then we can see that as ET increases: $E_{2}^{V}$ and $E_{1}^{R}$ coexist → $E_{B}^{2}$ exists → $E_{2}^{R}$ and $E_{1}^{R}$ coexist → $E_{B}^{1}$ exists → $E_{2}^{R}$ and $E_{1}^{V}$ coexist. Thus it is evidenced that ET plays a key role in the analyses of bifurcations of the Filippov system (5).

### 5.2 The global stability of equilibria

**Fig 2:**
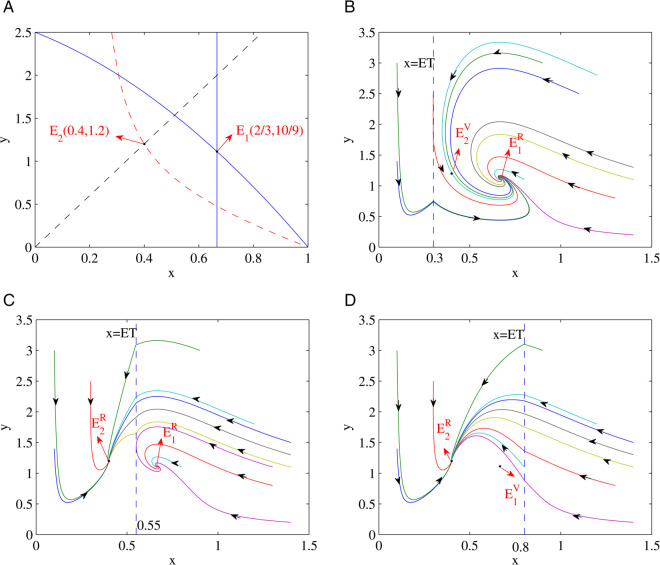
Illustrates the dynamical behavior of the Filippov system (5) which is plotted by using parameters $a_{1}=0.4,b_{1}=0.5,c_{1}=0.5,e_{1}=2,D_{1}=1$: (a) Shows the existence of the interior equilibrium point (in solid blue plot) for subsystem (1), the dotted curves (in red and black) are the interior equilibrium point of subsystem (2). (b) *E*_1_ becomes the only real equilibrium point and all solutions tend to converge into *E*_1_ eventually. (c) Both *E*_1_ and *E*_2_ are the real equilibrium points and all solutions tend to converge either into *E*_1_ or *E*_2_ eventually and (d) *E*_2_ becomes the only real equilibrium point and all solutions tend to converge into *E*_2_ eventually.

**Fig 3:**
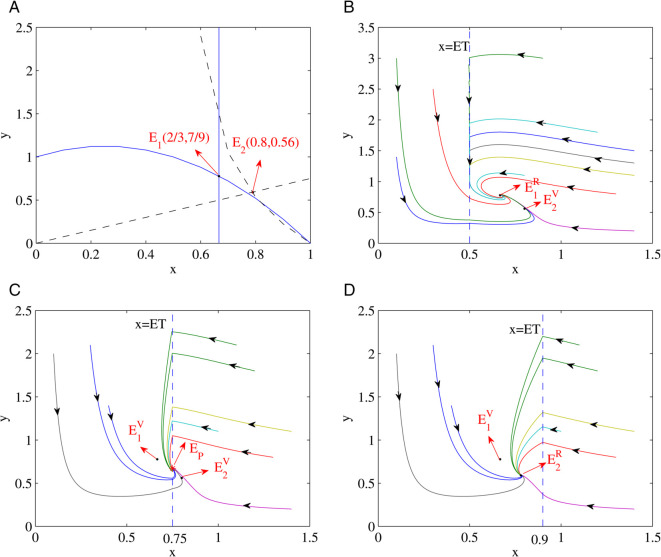
Illustrates the dynamical behavior of the Filippov system (5) similar to the above figure but using different parameters $a_{1}=1,b_{1}=2,c_{1}=2,e_{1}=3.5,D_{1}=1$: (a) Shows the existence of the interior equilibrium point for subsystem (1) in solid blue plot, and the interior equilibrium point of subsystem (2) in red and black lines. (b) *E*_1_ becomes the only real equilibrium and all solutions tend to converge into *E*_1_ eventually. (c) Both *E*_1_ and *E*_2_ are the virtual equilibrium points and all solutions tend to converge away from *E*_1_ or *E*_2_ eventually and to form a pseudo equilibrium *Ep* instead. (d) *E*_2_ is the only real equilibrium and all solutions tend to converge into *E*_2_ eventually.

The global stability of the equilibria in the Filippov system (5) is validated in this section through numerical simulation. In here we consider two cases of when $E_{1}=(x_{1}^{*},y_{1}^{*})$ and $E_{2}=(x_{2}^{*},y_{2}^{*})$:

(i) When the parameters are set as $a_{1}=0.4,b_{1}=0.5,c_{1}=0.5,e_{1}=2,D_{1}=1$ it can be shown through simple calculation that $x_{1}^{*}=\frac{2}{3}$ and $x_{2}^{*}=0.4$ indicating that $x_{1}^{*}>x_{2}^{*}$. When ET is varied the interior equilibrium points *E*_1_, *E*_2_ and pseudo-equilibrium *E*_*p*_ changed as shown in Fig 2(a). For example when $ET=0.3<x_{2}^{*}<x_{1}^{*}$, then *E*_1_ becomes $E_{1}^{R}$ and *E*_2_ becomes $E_{2}^{V}$ such that *E*_1_ becomes the only real equilibrium point where all solutions tend to converge into *E*_1_ eventually, as it is shown in Fig 2(b). When $x_{2}^{*}<ET=0.5<x_{1}^{*}$, then *E*_1_ is still $E_{1}^{R}$ and *E*_2_ becomes $E_{2}^{R}$. In this case both *E*_1_ and *E*_2_ are in real equilibrium and all solutions tend to converge into either *E*_1_ or *E*_2_ eventually, as it is shown in Fig 2(c). When $x_{2}^{*}<x_{1}^{*}<ET=0.8$, *E*_1_ becomes $E_{1}^{V}$ and *E*_2_ becomes $E_{2}^{R}$. Then *E*_2_ is the only real equilibrium in this case and all solutions tend to converge into *E*_2_ eventually as it is shown in Fig 2(d).

(ii) Similarly let’s set the parameters $a_{1}=1,b_{1}=2,c_{1}=2,e_{1}=3.5,D_{1}=1$ and it can be shown by simple calculation that $x_{1}^{*}=\frac{2}{3}$ and $x_{2}^{*}=0.8$ which indicates that $x_{1}^{*}<x_{2}^{*}$. As value of the ET is increased or decreased the interior equilibrium point *E*_1_, *E*_2_ and pseudo-equilibrium *E*_*p*_ are subsequently affected as shown in Fig 3. For example when $ET=0.5<x_{1}^{*}<x_{2}^{*}$, then *E*_1_ and *E*_2_ becomes $E_{1}^{R}$ and $E_{2}^{V}$ respectively. It can be seen that *E*_1_ becomes the only real equilibrium and all solutions tend to converge into *E*_1_ eventually as shown in Fig 3(b).When $x_{1}^{*}<ET=0.75<x_{2}^{*}$, *E*_1_ still exists as $E_{1}^{V}$ and *E*_2_ becomes $E_{2}^{V}$. Then *E*_1_ and *E*_2_ are both the virtual equilibrium, we can clearly see that all solutions tend to deviate from both *E*_1_ and *E*_2_ and tend to converge into the pseudo equilibrium *Ep*, which exhibits as the global asymptotic stability as shown in Fig 3(c). However when $x_{1}^{*}<x_{2}^{*}<ET=0.9$, then *E*_1_ becomes $E_{1}^{V}$ and *E*_2_ becomes $E_{2}^{R}$. In this case *E*_2_ becomes the only real equilibrium and we can see that all solutions tend to converge into *E*_2_ eventually as depicted in Fig 3(d).

## 6 Conclusions and biological significance

Nowadays, the Filippov system has been found to be useful to describe the real-world problems and investigated in many fields, such as those in physics, ecology and many other multidisciplinary subjects like networked control systems, multi-agent systems, neural networks, mechanical systems as well as in the integrated pest management for the ecosystem modelling etc [6–23,24]. Despite great deal of reports on non-smooth Filippov predator-prey system [6–23,24], none of them have ever considered using functional responses together with the Filippov system simultaneously. This work explores the behavior of the Filippov predator-prey system by imposing threshold policy on the population of prey for the initiation of a mixture of functional response types to study the dynamic of the ecosystem for the very first time. In this study the Holling-II and ratio functional responses have been implemented in the model depending on the population of the prey. For example, the mutual interference of the predator will play an important role when the number of prey is below the economic threshold (ET), thus the ratio functional response has been adopted in this work to model the ecosystem by taking into account of the competition among the predators. On the other hand when the population of the prey reaches or exceeds ET, the mutual interference among predators becomes negligible thus the Holling-II functional response has been selected in this work to model the dynamics of the ecosystem.

So we make use of Filippov theories and qualitative techniques with numerical simulations to investigate dynamical behaviors of proposed system in detail, including global dynamics of subsystems, the existence of sliding mode and different types of equilibria, sliding mode dynamics and the global stability of equilibria.

Stability analysis (asymptotic analysis) provides critical support for integrated pest management (IPM) by studying the equilibrium states of systems under long-term dynamics [3–5,23,24]. By establishing Differential Equations, the asymptotic stability of the pest-predator-crop system is analyzed to predict the long-term equilibrium points after reducing chemical pesticide use. For example, predator-prey model in [23] demonstrated that when negative feedback mechanisms exist in the system, asymptotic stability can suppress pest population outbreaks.

Furthermore, the proposed Filippov system together with the switchable functional response had been validated through numerical simulations. It has been demonstrated that the real equilibrium and pseudo-equilibrium points can coexist when the population of the prey is less than that of the predator (i.e. $x_{1}^{*}<x_{2}^{*}$ ); and in the case of when the population of the prey is more than that of the predator (i.e. $x_{1}^{*}>x_{2}^{*}$), only the virtual equilibrium and pseudo-equilibrium can coexist. As the economic threshold (ET) increases and when $x_{1}^{*}>x_{2}^{*}$, then the following equilibrium sequences can coexist: $E_{2}^{V}$ and $E_{1}^{R}$ coexist → $E_{2}^{R}$ and $E_{1}^{R}$ coexist → $E_{2}^{R}$ and $E_{1}^{V}$ coexist, as explicitly depicted in Fig 2 above. Since both *E*_1_ and *E*_2_ are both the real equilibrium, all solutions tend to either *E*_1_ or *E*_2_ eventually. This is an extremely interesting bistability phenomenon that can be seen in this switchable Filippov system. Similarly, when the ET increases and $x_{1}^{*}<x_{2}^{*}$, then the following equilibrium sequences can coexist: $E_{2}^{V}$ and $E_{1}^{R}$ coexist → $E_{2}^{V}$ and $E_{1}^{V}$ coexist → $E_{2}^{R}$ and $E_{1}^{V}$ coexist, as it is demonstrated in Fig 3 above. According to our results it has also shown that the sliding and escaping regions cannot coexist under our proposed system. In particular, it is noted that all trajectories of the prey and predator’s population are eventually converging into certain equilibrium points as it is demonstrated in the numerical simulation in Sect 5. This implies that there exists global asymptotic stability of equilibrium points under the proposed system, in which the population of preys eventually reaches a steady state of density at the real equilibrium and pseudo-equilibrium points. This means we don’t need to take any action at this point.

This work also highlights the significant role of the threshold ET in the process of pest controls.So the reasonable control threshold (ET) can be effective for prevention and control of pests. Consequently, our findings are valuable for how to draw up strategies effectively and when to take measures.This paper enriches the theoretical and methodological framework of future system dynamics modeling, holding significant theoretical and practical implications.

## References

[1] Lu X, Zhang Y, Yang L. Global boundedness and asymptotic stability for a food chain model with nonlinear diffusion. J Math Phys. 2024;65:101506.

[2] Zhang L, Li X. Spatial dynamics of a diffusive susceptible-infectious-recovered-susceptible epidemic model with transfer from infectious to susceptible. J Math Phys. 2025;66:011506.

[3] Liu J, Hu J, Yuen P. Extinction and permanence of the predator-prey system with general functional response and impulsive control. Appl Math Model. 2020;88:55–67.

[4] Hu J, Liu J, Yuen P, Li F, Deng L. Modelling of a seasonally perturbed competitive three species impulsive system. Math Biosci Eng. 2022;19(3):3223–41. doi: 10.3934/mbe.2022149 35240828

[5] Liu J, Hu J, Yuen P, Li F. A seasonally competitive M-prey and N-predator impulsive system modeled by general functional response for integrated pest management. Mathematics. 2022;10(15):2687. doi: 10.3390/math10152687

[6] Corts Garca C. Bifurcations on a discontinuous Leslie-Grower model with harvesting and alternative food for predators and Holling II functional response. Commun Nonlinear Sci Numer Simul. 2023;116:106800.

[7] Li W. Bifurcation analysis of a Filippov predator–prey model with two thresholds. Nonlinear Dyn. 2024;112(11):9639–56. doi: 10.1007/s11071-024-09527-6

[8] Li W, Chen Y, Huang L, Wang J. Global dynamics of a filippov predator-prey model with two thresholds for integrated pest management. Chaos, Solitons & Fractals. 2022;157:111881. doi: 10.1016/j.chaos.2022.111881

[9] Jiao X, Li X, Yang Y. Dynamics and bifurcations of a Filippov Leslie-Gower predator-prey model with group defense and time delay. Chaos, Solitons & Fractals. 2022;162:112436. doi: 10.1016/j.chaos.2022.112436

[10] Qin W, Tan X, Tosato M, Liu X. Threshold control strategy for a non-smooth Filippov ecosystem with group defense. Applied Mathematics and Computation. 2019;362:124532. doi: 10.1016/j.amc.2019.06.046

[11] Jiao X, Yang Y. Rich dynamics of a Filippov plant disease model with time delay. Communications in Nonlinear Science and Numerical Simulation. 2022;114:106642. doi: 10.1016/j.cnsns.2022.106642

[12] Zhou H, Tang S. Bifurcation dynamics on the sliding vector field of a Filippov ecological system. Appl Math Comput. 2022;424:127052.

[13] Zhang X, Tang S. Existence of multiple sliding segments and bifurcation analysis of filippov prey-predator model. Appl Math Comput. 2014;239:265–84.

[14] Arafa AA, Hamdallah SAA, Tang S, Xu Y, Mahmoud GM. Dynamics analysis of a Filippov pest control model with time delay. Communications in Nonlinear Science and Numerical Simulation. 2021;101:105865. doi: 10.1016/j.cnsns.2021.105865

[15] Hu C, Yu J, Chen Z, Jiang H, Huang T. Fixed-time stability of dynamical systems and fixed-time synchronization of coupled discontinuous neural networks. Neural Netw. 2017;89:74–83. doi: 10.1016/j.neunet.2017.02.001 28364661

[16] Deng J, Tang S, Shu H. Joint impacts of media, vaccination and treatment on an epidemic Filippov model with application to COVID-19. J Theor Biol. 2021;523:110698. doi: 10.1016/j.jtbi.2021.110698 33794286 PMC8007528

[17] Arafa AA, Hamdallah SAA, Tang S, Xu Y, Mahmoud GM. Dynamics analysis of a Filippov pest control model with time delay. Communications in Nonlinear Science and Numerical Simulation. 2021;101:105865. doi: 10.1016/j.cnsns.2021.105865

[18] Chong NS, Dionne B, Smith R. An avian-only Filippov model incorporating culling of both susceptible and infected birds in combating avian influenza. J Math Biol. 2016;73(3):751–84. doi: 10.1007/s00285-016-0971-y 26865385

[19] Zhu Y, Zhang Z, Ji J. Sliding dynamics of a Filippov ecological system with nonlinear threshold control and pest resistance. Communications in Nonlinear Science and Numerical Simulation. 2024;135:108052. doi: 10.1016/j.cnsns.2024.108052

[20] Gao C, Qiao S, An X. Global multistability and mechanisms of a memristive autapse-based Filippov Hindmash-Rose neuron model. Chaos, Solitons & Fractals. 2022;160:112281. doi: 10.1016/j.chaos.2022.112281

[21] Dong C, Xiang C, Xiang Z, Yang Y. Global dynamics of a Filippov epidemic system with nonlinear thresholds. Chaos, Solitons & Fractals. 2022;163:112560. doi: 10.1016/j.chaos.2022.112560

[22] Liu Y, Yang Y. Impact of non-smooth threshold control on a reaction–diffusion predator–prey model with time delay. Nonlinear Dyn. 2024;112(16):14637–56. doi: 10.1007/s11071-024-09796-1

[23] Zhou H, Zhang Q, Tang S. Qualitative analysis of the sliding vector field in a Filippov food chain model with integrated pest management strategy. Appl Math Comput. 2025;490:129188.

[24] Yu N, Zhang X. Complex dynamics in tick-borne disease transmission: a Filippov-type control strategy model with multiple time delays. Chaos, Solitons & Fractals. 2024;189:115673. doi: 10.1016/j.chaos.2024.115673

